# The Influence of Social Parameters on the Homing Behavior of Pigeons

**DOI:** 10.1371/journal.pone.0166572

**Published:** 2016-11-15

**Authors:** Julia Mehlhorn, Gerd Rehkaemper

**Affiliations:** Research Group “Comparative Neurobiology and Evolutionary Research”, Institute of Anatomy I, University of Duesseldorf, Germany; Bowling Green State University, UNITED STATES

## Abstract

Homing pigeons develop preferred routes when released alone several times from the same site, but they sometimes diverge from their preferred route when subsequently released with another pigeon. Additionally, group flights show a better homing performance than solo flights. But this knowledge is based on studies involving both sexes and lacks analyses of social parameters such as mating or breeding status, even though it is known that such parameters have an influence on behavior and on motivation for specific behavioral patterns. GPS trackers were used to track 24 homing pigeons (9 breeding pairs and 6 unmated females) as they performed a familiar 10km route in various pair and group combinations. Comparisons of efficiency indices (quotient between straight-line distance and pigeon’s track) reveal that unmated females show the best efficiency in single flights. Generally, group flights show the best efficiency followed by pair flights with a social partner of the opposite sex. Pair flights with the mated partner exhibit the poorest performance. Additionally, just before squabs hatching, females show a higher efficiency index when released at 8 am, compared to releases at 2 pm. Our results indicate that homing flight efficiency can provide insight into individual motivation and that social parameters have an influence on homing performance on a familiar route.

## Introduction

Homing pigeons are well-known for their excellent homing abilities, and the mechanisms they use to navigate homeward from distant release sites have been well studied. Several orientation cues and mechanisms e.g. olfactory cues, visual landmarks, sun compass, and magnetic compass are known to be involved in homing behavior, and parameters such as motivation and experience are also known to be important for fast and successful homing [1–6].

In recent years, the number of publications on homing tracks or pathways used by homing pigeons has greatly increased, which is due in large part to the advent of micro-global positioning system (GPS) data loggers that can be attached to a pigeon’s back, thereby enabling detailed analysis of flight trajectories [7,8]. The preferred routes of homing pigeons are rarely efficient straight lines, and there are differences between homing tracks from unfamiliar release sites and familiar release sites [9]. It is not entirely clear what makes a route from familiar sites preferred by pigeons, but Lipp et al. [10] have suggested that at least railway tracks and highways were used for following on the way home from familiar release sites. Yet there are regional differences, and this phenomenon is not observed in all pigeons [11, 12]. The fact is that homing pigeons develop individual preferred routes when released alone several times from the same site [13]. It must be considered that pigeons live in social groups, usually as monogamous pairs, and prefer to home in the company of other pigeons [14]. Thus, the presence or absence of conspecifics could influence the homing track and homing speed. If two pigeons with different preferred homing routes from the same familiar site are released together from that site, they come into conflict what to do. If the difference between two birds’ directional preferences is small, they average their routes, but if the difference surpass a critical threshold, either the pair splits apart or one of the two birds become the leader [15]. Several research groups have also shown that the homing performance of two or more birds was more efficient than the homing of either one, even when single performance of individual birds was poorer [15, 16, 17].

All this knowledge about the homing tracks used is mostly generalized for both sexes, males and females, and does not include social parameters such as mating or breeding status. Homing pigeons live in flocks with a flexible social structure, not a strict hierarchy. They live in monogamous pairs and usually mate for life. They have fixed territories centered on a nest and males show strongly territorial behavior, but both birds of a well-bonded pair will defend the nest territory. Sometimes, particularly dominant males try to mate with more than one female. Males and females both engage in breeding and the manner in which the sexes share incubation is ritualized. Males sit from mid-morning (approximately 10 am) to late afternoon (approximately 5 pm), females from late afternoon to mid-morning the next day [18]. Pigeon fanciers have used these particularities of behavior to develop differential strategies aimed at improving the motivation to home when displaced and the homing performance during competition. One strategy is that they allow the pigeons to mate and to build a nest, but then they separate at least one partner from the nest for a few days. After returning from a race, these pigeons are now allowed to go directly to their waiting partner and nest. Another, apparently successful, method is to bring a possible competitor into the loft just before catching the pigeons for a race. This motivates the pigeons to hurry home [19].

This anecdotal knowledge and the high number of studies of homing tracks without any analysis of social parameters leads to the question of what ways parameters such as sex, mating status, and/or breeding status influence the homing performance or the motivation to fly home. It is better to analyze homing tracks for a familiar route, because it makes the performance mostly independent of the individual pigeon’s navigational ability. If every pigeon knows the way home, any differences in homing performance should be due to other reasons. Biro et al. [15] have provided an experimental design for this. They analysed the homing tracks of solo and dual flights on a familiar route. In our study we also used high-resolution GPS loggers and adopted Biro’s et al [15] experimental design as far as possible. But we put our focus on how social parameters such as sex, mating status, and breeding status might influence homing performing a familiar route. We anticipated that mating and/or incubation functions as a motivational factor for homing performance. Additionally, we made group flights to investigate whether flying in groups also increased performance.

## Materials and Methods

### Ethics Statements

All applicable international, national, and/or institutional guidelines for the care and use of animals were followed. The study was approved by the Committee on the Ethics of Animal Experiments of North Rhine-Westphalia (Ref. 84–02.04.2011.A137).

### Study Setting

Nine well-established pairs of homing pigeons (*Columba livia* f.d., 9 males and 9 females) and six unmated females (unmated males were not available) from the neighboring loft were selected as subjects. The two lofts were separated from each other by just a small corridor, and both lofts were constructed and equipped in the same way. All the pigeons were at least one year old and successful homers over distances up to 300 kilometers. Males and females of mated pairs were separated for several weeks and brought together just shortly before the beginning of the tests in order to synchronize their breeding cycles. They were allowed to lay eggs and to breed (to ensure that mating succeeded). Food and water was available ad libitum. All pigeons were housed in the pigeon lofts of the University of Düsseldorf, with breeding boxes and seating-accommodations inside. The experiments were carried out during the summertime (August-September). The weather was always sunny or moderately cloudy with temperatures between 17–24°C. The wind speed and wind directions according to the “German Weather Service” can be seen in the supporting information, S1 Table. Releases were done before the beginning of main molting. The pigeons of both groups had just changed a few of their primary feathers.

All pigeons were fitted with a Velcro strip, attached to their back with leather glue applied to trimmed feathers, and they were trained to carry plexiglas dummies (weight: 18 g). Each bird was then released alone from the same release site 8 times consecutively, with one release per day (straight-line distance ~10 km, flight direction south-southeast). All mated females had laid their eggs by the end of this training period. After that, the pigeons were tagged with GPS loggers (12g; Technosmart; Rome, Italy) for each flight and had to fly the same route again for 6 times, consecutively once per day. GPS loggers (see [7] for technical description) were also attached to the pigeons’ backs by the Velcro strips. All releases were carried out from release boxes which had an exit on one side and permitted a view of the sky. By the end of this training phase, we assumed that the pigeons were quite familiar with this release site and the way home.

Having completed these solo flights, the pigeons were assigned to different pair and group combinations and released from the same site simultaneously with their flight partner(s). Mated pigeons had to fly first with their mated partner (I) and then with a fellow of the opposite sex that was not their own mating partner (II). In the next release, pigeons had to fly with a pigeon of the same sex (III). Subsequently, we released groups of six pigeons consisting of 3 mated pairs (IV). The unmated females also carried out the three flights in pairs, matched up with one another and all six unmated females were released together for the group flight. As in [15], birds were given one or two solo flights after each paired release, in order to remember their own preferred route. Each flight unit (solo pigeon, duo, or group) was not released until the previous flight unit was no longer visible to the researcher and a minimum of 10 minutes had elapsed. The last releases were group flights with all 9 mated males together or all 9 mated females together. While previous releases were randomly assigned during the day, these final flights in groups of 9 had release times of 8am and 2pm. Each group made one flight at 8 am and one flight at 2 pm. The reason for choosing these release times was that males and females have different, fixed times of the day for their shift sitting on the eggs, as mentioned above. The male sits on the nest from approximately 10am to 5pm; whereas, the female sits on the nest for the rest of the time. Thus, at 8 am, the females should be on the nest, and at 2 pm, the males should be there. These last releases took place approximately 17 days after egg laying. Since pigeons have a breeding period of approximately 18 days, the squabs would be hatching quite soon. We assumed that this anticipated parenting could increase their motivation for efficient homing flight.

The constellation of duo and group flights was randomized. To prevent any influence of different weather conditions or environmental effects, we disclaimed randomizing the order of pair and group flights.

Time-stamped positional fixes were logged every second and were downloaded upon recovery of the device. The positional data were superimposed onto Google Maps using Habitat Tracker software (Biobserve; St. Augustin, Germany). To minimize observer bias, blinded methods were used whenever behavioral data was recorded. Random numbers were assigned to each pigeon, in order to prevent the analyst from knowing which data set belonged to which pigeon until the end of the analysis.

### Data Analysis

For each flight, track length was calculated as the total distance travelled to reach home (the sum of the distances separating all consecutive points of a track). Calculating track length started promptly after releasing and ended once the pigeon reached the university building with the home loft. An efficiency index was calculated as the straight-line distance between the release site and home, divided by the individual track length. Since an efficiency index of 1 would mean that the pigeon flew a straight-line, values close to 1 show a high homing efficiency. We used the efficiency index and not the homing time or homing speed for analysis because homing time depends in part on the physical strength (and thus the flight velocity) of the individual pigeons, which was of no interest in this study on the directness of the flight route. To prove this argument, we compared at first body weights of male and female pigeons statistically by t test. To exclude a correlation between wind speed/wind direction and homing efficiency, Pearson Product Moment Correlations were carried out. Nevertheless, averaged homing speed values for each track (straight-line distance divided by the homing time) were also provided and some calculations were also carried out with them.

Efficiency indices were compared statistically by t test, Mann-Whitney test (in case of non-normal distribution), ANOVA (on ranks), or Friedman Repeated Measures Analysis of Variance on Ranks (FRM ANOVA on ranks) in case of comparisons between the sexes. Comparisons within the sexes were done with parametric and non-parametric tests for dependent data, namely the paired t test and Wilcoxon Signed Rank test. The level of significance was 5%. SigmaPlot/SigmaStat version 12.0 was used for all statistical calculations.

## Results

We first compared body weights of males and females and noticed that there were significant differences between them with a physical superiority for the males (498.148 g ± 49.54 vs 461.500 g ± 63.97; t test, t = 2.215, p = 0.032). There were no significant correlations between the homing efficiency and the wind speed (Pearson Product Moment Correlation, r = 0.335, p = 0.344) or wind direction (r = -0.555, p = 0.096). This is the same between homing speed and wind speed (r = 0.227, p = 0.529) or wind direction (r = -0.363, p = 0.303).

After this we examined the development of homing routes during repeated solo flights from the same release site. All pigeons improved their efficiency during these flights. Individual efficiency index increased significantly in the first five days of training with GPS loggers (FRM ANOVA on ranks, Chi-square = 21.567, p<0.001; Table 1). After that, the efficiency stagnated or even decreased. This was the same after analyzing speed values (FRM ANOVA on ranks, Chi-square = 42.567, p<0.001; Table 1) what makes it likely, that both parameter, efficiency index and homing speed, would give similar results. Because of the found differences between body sizes of the sexes, we kept the use of efficiency indices and carried out the following analysis just with them.

**Table 1:** Efficiency indices and speed of all pigeons for solo flights with GPS-loggers (mean ± sd).

	Efficiency index (n = 24 per day)	Speed (km/h, n = 24 per day)
Day 1	0.784±0.107	53.410±7.183
Day 2	0.815±0.131	61.239±7.327
Day 3	0.852±0.096	61.573±7.688
Day 4	0.870±0.075	62.597±5.669
Day 5	0.899±0.057	70.300±9.185
Day 6	0.870±0.0469	53.930±7.047
Day 7	Duo flight I	Duo flight I
Day 8	0.790±0.0198	53.917±7.047
Day 9	Duo flight II	Duo flight II
Day 10	0.865±0.0846	63.600±9.345
Day 11	Duo flight III	Duo flight III
Day 12	0.828±0.116	72.548±13.809
Day 13	Group flights	Group flights

**Fig 1:**
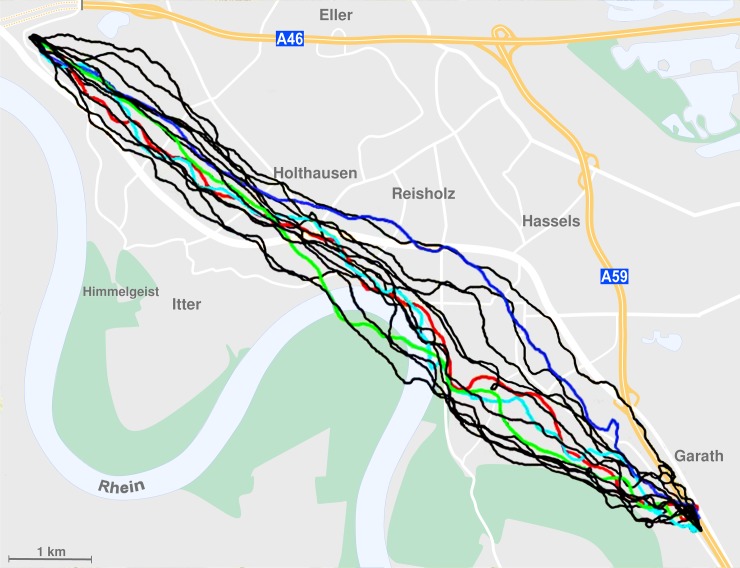
Map showing all solo, duo, and group flights of an unmated female. Black = solo flights; red, blue, green = 1^st^, 2^nd^, and 3^rd^ duo flights; light blue = group flight.

Next we analyzed solo flights of our three groups of pigeons (Table 2). For statistical comparison, we used the efficiencies of all solo flights: the six ones before the beginning of the pair and group flights and the single flights between them. Here we noticed that there were significant differences between the three groups (ANOVA on ranks, H = 8.421, p = 0.015, Table 2). Unmated females had the highest efficiency in single flights (0.860±0.103, mean±sd), followed by the mated males (0.831±0.104). Mated females had the poorest efficiency (0.815±0.104). Mated females also flew slower (58.358±10.022) than both other groups (mated males: 60.649±11.038; unmated females: 62.743±8.590), but not significantly so (ANOVA on ranks, H = 5.937, p = 0.051, Table 2). Fig 1 illustrates an example of GPS tracks obtained from an unmated female.

**Table 2:** Efficiency indices and homing speed of all flights of all pigeons (mean ± sd).

	Efficiency	Speed (km/h)
**Solo flights**–unmated females	0.860±0.102	62.743±8.590
**Solo flights**–males	0.831±0.104	60.649±11.038
**Solo flights**–mated females	0.815±0.104	58.358±10.022
**Duo flights:**		
Mated pairs	0.795±0.044	64.884±6.586
Duos of same sex–unmated females	0.891±0.070	70.958±11.570
Duos of same sex–males	0.803±0.095	56.703±6.963
Duos of same sex–females	0.827±0.037	58.434±5.097
Duos of opposite sex	0.910±0.023	81.956±5.292
**Group flights** (unmated females)	0.956±0.009	91.534±1.842
**Group flights** (mated pairs)	0.949±0.018	91.278±3.3655

See the main text and Table 3 for statistical results. (The means for solo flights are calculated from all solo flights).

**Fig 2:**
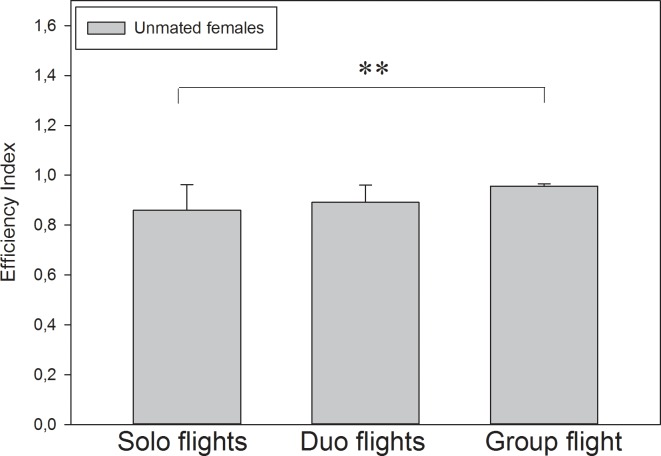
Efficiency indices (mean ± sd) of unmated females in solo, duo, and group flights (**p = 0.009).

No pigeon stopped on their way home in any of the solo, duo or group flights. Besides, the pigeons never split apart in any of the duo or group flights; they always remained together. No circling over the releasing site could be observed. A comparison of the solo, duo, and group flights of unmated females shows that efficiency increases with group size in a significant way (ANOVA on ranks, H = 9.420, p = 0.009; Fig 2). The group flight (consisting of all six unmated females) showed the highest efficiency (0.956±0.009) followed by duo flights with another unmated female (mean efficiency index: 0.891±0.070). Single flights showed the poorest efficiency (0.860±0.103).

**Fig 3:**
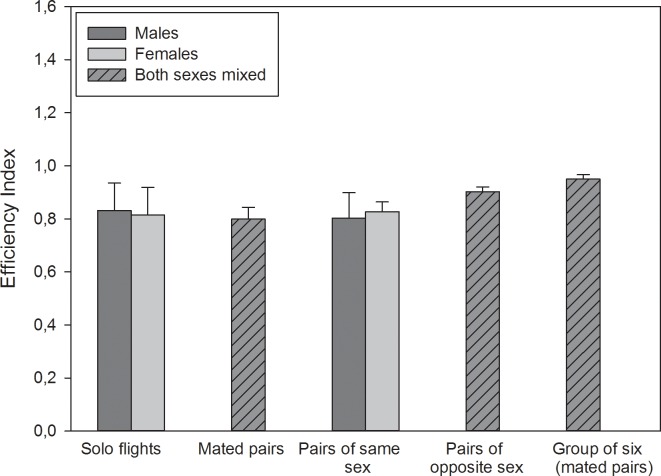
Efficiency indices (mean ± sd) of mated males and females in solo, duo, and group flights. See the main text and Table 3 for statistical results.

Fig 3 and Tables 2 and 3 summarize the results of the mated pigeons; Table 2 also provides an overview of homing speed. In combination I (“mated pairs”, Fig 3) and the group flights (“group of six”, Fig 3) males and females are presented with one vertical bar because they always flew together and therefore always had the same efficiency. There was a statistically significant difference between the different flight combinations (ANOVA on ranks, H = 51.452, p<0.001). Both sexes showed the lowest efficiency in duo flights with their partner (0.795±0.044) and the highest efficiency in group flights with two more pairs (0.949±0.018). The efficiency index of the group flight was always significantly higher than the efficiency index of solo or duo flights (see Table 3). Duo flights with a social partner of the opposite sex showed a significantly higher efficiency than duo flights with a social partner of same sex (paired t test, males: t = 3.184, p = 0.013; females: t = -7.552 p<0.001) or the mated partner (paired t test, males: t = 6.457, p<0.001; females: t = 7.526, p<0.001). Females also showed a significantly higher efficiency index in duo flights with a social partner of the opposite sex than in solo flights (Mann Whitney test, T = 531.50, p = 0.002).

**Table 3:** Statistical results of comparisons between efficiency indices of solo, duo, and group flights (n.s.: p>0.05).

	Solo flights -males-	Solo flights -females-	Duo flight I -males-	Duo flight I -females-	Duo flight II -males-	Duo flight II -females-	Duo flight III -males-	Duo flight III -females-	Group flights
Solo flights -males-	/	n.s.	n.s.	T = 252.00, p = 0.032	n.s.	n.s.	n.s.	T = 493.0, p = 0.007	T = 1096.5, p<0.001
Solo flights -females-		/	n.s.	n.s.	n.s.	T = 531.5,p = 0.002	T = 347.0,p = 0.030	n.s.	T = 1146.0, p<0.001
Duo flight I -males-			/	/	t = 6.457, p<0.001	t = -6.547, p<0.001	n.s.	n.s.	t = 8.353, p<0.001
Duo flight I -females-				/	T = 45.00,p<0.001	t = 7.526, p<0.001	n.s.	n.s.	t = -9.658, p<0.001
Duo flight II -males-					/	n.s.	t = 3.184, p = 0.013	t = -3.070, p = 0.008	t = -5.622, p<0.001
Duo flight II -females-						/	t = 4.000, p = 0.001	t = -7.552, p<0.001	n.s.
Duo flight III -males-							/	n.s.	Z = -2.668, p = 0.004
Duo flight III -females-								/	Z = 2.692, p = 0.004

Duo flight I: mated pairs; Duo flight II: pairs of opposite sex; Duo flight III: pairs of same sex; Group flights: three mated pairs.

**Fig 4:**
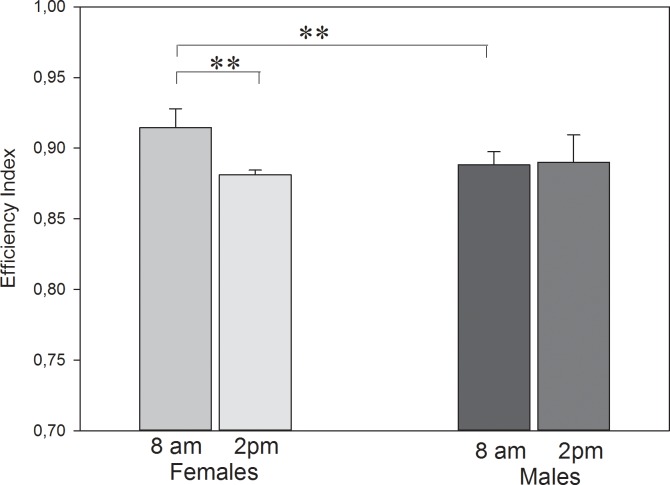
Efficiency indices (mean ± sd) of mated females and males at various times of the day (**p = 0.008).

The results of the releases at different times of the day are shown in Fig 4. Mated females showed a significant higher efficiency at 8 am than at 2 pm (0.914±0.013 vs. 0.881±0.004; Wilcoxon Signed Rank test, T = 43,000, p = 0.008) and in comparison to the mated males at 8 am (0.888±0.012; Wilcoxon Signed Rank test, T = -36,000, p = 0.008). Mated males did not show a significant difference between 8 am and 2 pm (0.888±0.012 vs. 0.890±0.021; Wilcoxon Signed Rank test, T = 15,000, p = 0.426) or in comparison to mated females at 2 pm (0.890±0.021 vs. 0.881±0.004, Wilcoxon Signed Rank test, T = 20,000, p = 0.195).

## Discussion

In this study we examined the influence of social parameters such as sex, mating status, or breeding status on homing performance for a familiar route in homing pigeons. After training the pigeons to a release site approximately 10km from their home loft, we released them in various duo and group constellations, to assess the social parameters mentioned. Due to repeated releases and the fact that the distance between the release site and the home loft was not very long, the pigeons became more and more familiar with this route, and very probably knew the most efficient route. The initial solo flights showed an increase in efficiency during the first days and then stagnation. Thus, we assume that training or learning would not have had any further influence on subsequent flights and that differences in the efficiency index were due to differences in motivation. Of course, group size can also improve efficiency, and generally it is difficult to distinguish between the ability to fly home and the motivation to make use of it, but it seems to be most probable that motivation influences efficiency index of a familiar route in a strong way.

We first showed that efficiency increased during the first five days of training with GPS loggers. On day six and in the single flights between the pair and group flights, efficiency stagnated or even decreased slightly. This is consistent with the findings of Guilford & Biro [9] who observed a gradual development of route performance with decreasing effects after a rapid initial improvement. This is also characteristic of learning processes in general.

Our data also confirms the results of several other research groups [13, 15, 20, 21] that repeated releases improve homing performance. Pigeons typically reach a high level of route efficiency within approximately eight to ten flights [13]. Apparently we observed a learning process whereby the birds were adopting the most efficient route home, which of course means an increasing approximation to the straight-line.

Generally, mean efficiency index of solo flights was similar to those described in the literature, where the values vary between 0.66 and 0.91, but mostly are around 0.83 (summarized in [9]). Theoretically, efficiencies seem to allow room for some improvement. Wiltschko et al. [12] discussed this point and suggested a certain increase in efficiencies with increasing distance. They assumed that the shortness of the distance (and a 10 km straight-line distance as in our study is in fact short for a homing pigeon) may not present a real challenge for the pigeons, so they may not have felt a need for further improvement. Our observed range between efficiencies supports our hypothesis that the efficiency index over a (relatively short) familiar route can be influenced by the social setting or the type of social relations to flight companions. Only a few studies have dealt directly with the possible influences of birds’ motivation on homing performance [22, 23].

Interestingly, unmated females showed a better efficiency index in both, solo flights and duo flights than the mated females or males. It seems to be that celibacy is not just a motivational factor for homing performance but even a strong one. The higher efficiency index of the unmated pigeons’ group flights is consistent with the findings of Biro et al. [15] and Dell’Arriccia et al. [16] which showed that group flights generally show a better homing performance than solo flights, even if the solo performance of the individual birds was poorer. Several research groups have described this phenomenon and explained it with the “many-wrongs” principle and other models of group navigation that predict the cancelling of individual navigational errors [15, 16, 24, 25]. By contrast, Santos et al. [26] showed that the circling time increases with group size because of establishing a leader. We did not observe this in our duo and group flights, and in our opinion, one must distinguish between the initial circling and the final chosen route, which represents the efficiency index. The efficiency index of mated pigeons’ group flights was higher as in solo or duo flights. Thus, group size has an effect on efficiency that is distinct from motivational effects. The speed values showed similar tendencies, but their interpretation is more difficult because we cannot estimate the influence of physical fitness in case of divergences (see above).

It is assumed that pigeons released alone show extended circling time at the release site because they search for other pigeons; whereas, duos and small flocks usually leave the release site much faster and more directly [27]. Thus, it must be expected that duo or group flights show a higher efficiency index than solo flights because of a socially motivated behavior at release site. This is consistent with our findings for the unmated females but not for the mated pigeons. Interestingly, the motivation to fly home via the most direct route is poorest if mated pairs fly together. Apparently, flying with the mated partner reduces motivation to go home, and thus the efficiency index, because staying with the mated partner is the motivational factor and acts as a reward. This is consistent with common treatments of pigeon fanciers who know that the mated partner in combination with the breeding place leads to a very high motivation in pigeons and can be used to improve homing performance. So usually just one pigeon from the couple participates in a pigeon race while the other one waits in the loft [19].

It would be interesting to test whether the phenomenon of poorer performance in (mated) pair flights disappears when flying longer distances or in flights from unfamiliar release sites, since these kinds of flights would be a bigger challenge for their navigational capability and it is possible that now non-navigational parameters become more dispensable. As mentioned above, the efficiency index increased significantly in group flights where three mated pairs were released together. There is always a tendency to travel in groups (even in flying a familiar route), and it has been shown that the nature of social relationships within the group can have an influence on the choice of route [28]. In our study, the good homing performance in all groups indicates that group flights are more efficient in general and not strongly influenced by sex, mating, or incubation.

Our last releases showed that breeding or incubation status is a motivational factor, but these differences are significant only in females, which seems to suggest that female pigeons attach greater importance to the behavior of sitting on eggs, perhaps because of their larger investment (they lay the eggs and have longer turns of breeding) [29]. Wallraff [6] showed that homing performance exhibits a clear annual periodicity (at least in birds released at unfamiliar locations), but he did not find a correlation between the breeding cycle and homing performance, maybe because domestic pigeons breed basically all year if e.g. food availability is guaranteed. Our results show that there is a correlation between the breeding status and the efficiency index. An existent clutch or an upcoming eclosion increases motivation to home, at least in females and at least in performing a short distance from a familiar site. This is consistent with the findings of Clausen et al. [22] who showed that the breeding cycle has an influence on homing (speed). In their study, the pigeons showed an increase of homing performance during incubation with a peak at eclosion time and in the first days with the squabs (because of psychological aspects (which means motivation) and hormonal changes). Indeed we made the 8am and 2pm releases just before the expected hatchling, but the pair and group flights were done a few days earlier and thus, not during the peak time. Lipp [30] also showed in his study about nocturnal homing that breeding pigeons and pigeons with a high level of courtship activity appeared to be much better motivated for homing than other pigeons.

We decided to randomize the constellation of the duo and group flights but not the order (see above). Of course, further experiments with a randomized flight order would be interesting to verify our results, but we did consciously decide to use the non-randomized order. Bell [31] has recently written that a randomized order of treatments is often the best approach for an analysis. Yet she also pointed out that there are also several advantages for a fixed order, e.g. that all individuals have the same experience at each treatment. Precondition should be the assumption that, if there is a carryover effect, it should be similar for all individuals, or if individuals do differ in the carryover effect, then the variation among individuals in the carryover effect would be small relative to the mean amount of the carryover effect. If we assume that the carryover effect in our study is based on experience (or training, or learning), this precondition is met. To exclude a possible carryover effect of incubation time or incubation status and to ensure similar conditions for duos and groups, we made our 8am and 2pm releases just before the expected hatching but the duo and group flights a few days earlier and not during the peak time of incubation. Of course, monitoring and timing the breeding status in an experimental loft requires a lot of work and attention, which may explain at least part of the common neglecting this factor in studies.

The difficulty of reproducibility makes homing difficult to analyze in general. Aperiodic fluctuations have been found in the course of hours, from day to day, and among different years. And of course there is a high dependency on a) the actual weather and b) the release site, or more exactly the terrain between the release site and home, with its characteristic landmarks, geomagnetic field etc. [32]. But nonetheless, analyzing homing tracks can deliver exciting insights into birds’ home finding processes. Our study give an insight in a special case (familiar route, short distance, nonstop flights) for which the efficiency index positively correlate with homing speed.

We would like to conclude by noting that our limited knowledge of pigeons’ motivation to home is not only a problem of the evaluation and interpretation of homing data obtained with normal untreated birds. Even more so, this problem is relevant for investigations that use birds that are experimentally manipulated in a particular way. If their performance is reduced, it is necessary to ask whether the experimental interference reduced their ability or their willingness to home.

## Supporting Information

S1 Table Wind speed and wind direction for releasing times. (DOCX)
