# Supplementary material for: The Influence of Social Parameters on the Homing Behavior of Pigeons
Source: PLoS One. 2016 Nov 15;11(11):e0166572. doi: 10.1371/journal.pone.0166572 (PMC5112789; doi:10.1371/journal.pone.0166572)
Supplement: S1 Table — (DOCX) [file pone.0166572.s001.docx]

**S1 Table.** **Wind speed and wind direction for releasing times**

|  | Wind speed (m/s) | Wind direction |
| --- | --- | --- |
| Day 1 (solo flight) | 6,3 | 230° |
| Day 2 (solo flight) | 3,3 | 210° |
| Day 3 (solo flight) | 1,7 | 210° |
| Day 4 (solo flight) | 1,6 | 310° |
| Day 5 (solo flight) | 3,1 | 180° |
| Day 6 (solo flight) | 8,0 | 240° |
| Duo flight I (mated pairs) | 8,3 | 220° |
| Duo flight II (pairs of opposite sex) | 6,6 | 170° |
| Duo flight III (pairs of same sex) | 6,0 | 240° |
| Group flight (mated pairs) | 7,4 | 170° |
